# The role of temperature in affecting carry-over effects and larval competition in the globally invasive mosquito *Aedes albopictus*

**DOI:** 10.1186/s13071-019-3391-1

**Published:** 2019-03-19

**Authors:** Nnaemeka F. Ezeakacha, Donald A. Yee

**Affiliations:** 1 School of Biological, Environmental, and Earth Sciences, University of Southern Mississippi, Hattiesburg, MS 39406 USA; 2 Present Address: Department of Entomology, Pennsylvania State University, 012A Chemical Ecology Laboratory, University Park, PA 16802 USA

**Keywords:** Climate change, Development time, Detritus, Ecology, Fecundity, Mosquitoes

## Abstract

**Background:**

Ectotherms, like mosquitoes, have evolved specific responses to variation in environmental conditions like temperature, and these responses could confer a fitness benefit or cost when carried-over to different life stages. However, effects of temperature on animals with complex life-cycles often only focus on part of their life-cycle, or only consider how single aspects of life-history may carry over to new stages. Herein we investigated how temperature affects intraspecific larval competition and carry-over effects from larval to adult stages in the widespread invasive Asian tiger mosquito *Aedes albopictus*.

**Methods:**

For larval competition, larvae were reared at three densities (10, 20, and 40 individuals) across three source temperatures (21 °C, 27 °C and 34 °C). To test carry-over effects, adult survival was measured for individuals crossed with adult temperatures of 21 °C, 27 °C and 34 °C from the larval density of 20 individuals at each source temperature. Fecundity data also were obtained from mated females.

**Results:**

For competition, there was a significant interaction between larval density and temperature, with the smallest females, who took the longest to develop, produced in the highest temperatures; density generally accentuated this effect. Regarding carry-over effects, adults exposed to higher temperatures lead to greater differences in fecundity and survival of adult populations.

**Conclusions:**

Temperature appears to affect life-history of developing larvae under competitive interactions and can also alter adult fitness as the disparity between larval rearing and adult habitat temperatures increases. This has importance for our understanding for how different life-history stages of *Ae. albopictus* and other vectors of disease may respond to changing climates.

## Background

Most multicellular organisms have distinct life stages that vary in size, morphology, physiology, and other measurable traits. Experiences from different seasonal environments, habitats, and microclimates often result in these life stages developing different physiological sensitivities and responses, thereby leading to differential contribution to overall lifetime fitness [1]. For many groups, there is growing evidence that life stages may be interdependent and affect each other across metamorphosis, such that individual characteristics and responses to environmental conditions in one life stage can influence their characteristics and responses in subsequent life stages [2, 3] and beyond the current generation [2]. Herein, we focus on an examination of the effects of drastic temperature shifts acting on the immature and adult stages, and any consequences these have for life-history performance in the globally invasive Asian tiger mosquito *Aedes albopictus* (Skuse).

Temperature is regarded as one of the most important abiotic environmental factors affecting biological processes and physiological functions, including locomotion, growth and reproduction in ectotherms. For example, individuals reared at higher temperatures may develop more rapidly compared to lower temperatures, but adults tend to be smaller [4] with reduced fitness given that size is often positively related to fecundity [5, 6]. In many insects temperature is especially important in determining life-history characteristics. However, many investigations of temperature effects on insects have usually focused on either the larval or terrestrial portion of the life-cycle, but not both simultaneously (i.e. carry-over effects). Here we define carry-over effects as those that are non-reversible, occurring from one life-history stage to the next within the same generation, and in contrast to those that occur between seasons for the same individual, often in vertebrates [7]. Mosquitoes are an important group of insects for their proclivity to spread pathogens that are agents of human and animal disease. In addition, mosquitoes are also representative of many insects with an aquatic larval phase followed by a terrestrial adult stage. Temperature has been shown to affect a number of life-history parameters in mosquitoes, including egg viability [8], larval development [9], blood-feeding behavior [10], female fecundity [11], adult longevity [6, 12], interactions with parasites and arboviruses [13, 14], wing size [15] and population sizes [16]. Despite the plethora of information relating temperature to mosquito biology, relatively few studies have evaluated the net effect of temperature on multiple traits and their carry-over effects across mosquito life-history stages.

Environmental conditions experienced during larval development have become increasingly recognized to have an important influence on adult mosquito life-history traits [17] and variation in quality of larval habitats could be carried over to affect adult life history. For instance, lower temperature and higher resource availability are often positively correlated with body size, and larger individuals often exhibit increased probability of survival, fecundity and overall fitness [4, 18]. The larval environment has also been demonstrated to shape mosquito vector competence by significantly affecting susceptibility to arboviruses [19] and parasites [20]. However, studies that have examined carry-over effects of conditions in the larval habitat on adult longevity, fecundity, or vector competence, have largely focused on effects of resource availability or sub-lethal insecticide exposure [20, 21].

The life stages of mosquitoes and other insects with complete metamorphosis occupy different habitats and shifts in microclimate experiences after transition between the immature and adult stages are common. As a result, temperatures acting on immature stages in aquatic habitats can interact with diel temperatures in terrestrial habitats of adult stages to affect adult phenotype [22]. Studies on carry-over effects of larval rearing temperature on adult characteristics have focused mainly on changes in vector competence [23, 24]. However, only recently have studies began to investigate the collective effects of temperatures experienced during larval and adult stages [22, 25, 26].

This study investigated the effects of temperatures on performance of larval and adult stages in the container-dwelling mosquito *Ae. albopictus. Aedes albopictus* is a world-wide invasive species native to Southeast Asia that is capable of transmitting numerous arthropod-borne viruses [27]. Since its introduction to the USA, the distribution and geographical range of *Ae. albopictus* has greatly expanded [28] mainly due to its competitive superiority over co-existing species and the important role temperature has played in its population dynamics and range expansion [29]. We designed two experiments to examine the effects of temperature on carry-over effects in *Ae. albopictus*. The first experiment tested the hypothesis that the temperature experienced during larval development alters the intensity of density-dependent intraspecific larval competition. Based on current knowledge, we predicted that there would be an interaction between larval density and temperature such that development times, survival, and emerging adult body mass would decrease with increasing temperatures and densities; evidence for this effect occurs across a wide range of taxa [30]. The second experiment tested the hypothesis that adult performance would vary with both larval habitat temperature and adult habitat temperature. Our prediction was that adult female fecundity and survival would be highest when larval and adult habitat temperatures were similar. We selected the specific response variables as these (i) have often been measured in other studies and thus would make the results of our study comparable; (ii) are relatively easy to measure under laboratory circumstances; and (iii) are those that could be linked to arbovirus infection and thus disease transmission [22–24].

## Methods

### Experiment 1: Temperature and intraspecific larval competition

Eggs of *Ae. albopictus* were obtained from F_1_ eggs produced by mosquitoes collected in and around Hattiesburg, MS, USA. Eggs were hatched synchronously in a solution of 0.33 g of Nutrient Broth (DifcoTM, BD, Sparks, MD, USA) and 750 ml of reverse-osmosis (RO) water following which all first-instar larvae were rinsed after hatching to remove nutrient broth. After 24 h, hatched first-instar larvae were randomly selected and placed in microcosms of 250 ml tripour plastic beakers containing 200 ml of reverse osmosis (RO) water. Larvae were reared at three densities: 10, 20, and 40 individuals. Microcosms were placed into trays with up to 20 microcosms per tray and all trays were placed into 3 different environmental chambers (Percival WE-35VL, Boone, IA) with 12:12 day: night cycle with 85% relative humidity. Here, three different larval rearing temperatures were used: 20 °C, 27 °C and 34 °C, representing low, medium and high temperatures required for mosquito performance [25]. Rearing temperature (3) and density (3) were crossed and replicated ten times for a total of 90 experimental units. Trays were rotated daily to control for effects of location within the environmental chambers. The food source for mosquito larvae consisted of 0.05 g of 50:50 lactalbumin-Brewer’s yeast for the first week after which 0.02 g were added every other week until pupation.

Microcosms were inspected daily for pupae that were removed when observed and transferred to 0.25 dram glass shell vials. The date of pupation, date of emergence and sex were recorded for each newly eclosed adult after which all adults were freeze-killed and dried for 48 h at 50 °C. Adult dry mass was measured to the nearest 0.0001 g using a XP2U ultra-microbalance (Mettler-Toledo Inc., Columbus, OH, USA). Survivorship (the percentage of initial larvae surviving to adulthood) was calculated for each replicate of each treatment combination. This experiment ran for 60 days after which mosquito larvae that had not pupated and pupae that did not eclose were counted as mortalities.

### Experiment 2: Adult performance with larval and adult habitat temperatures

Similar egg hatch and larval rearing procedures from the experiment described above on temperature and larval competition were used for this experiment but with the following changes: (i) only one larval density (20 individuals) was used; (ii) density was replicated 20 times for each of the three rearing temperatures, for a total of 60 experimental units; and (iii) emerging adults were not freeze-killed but kept alive for fecundity and survival assessments.

**Fig. 1:**
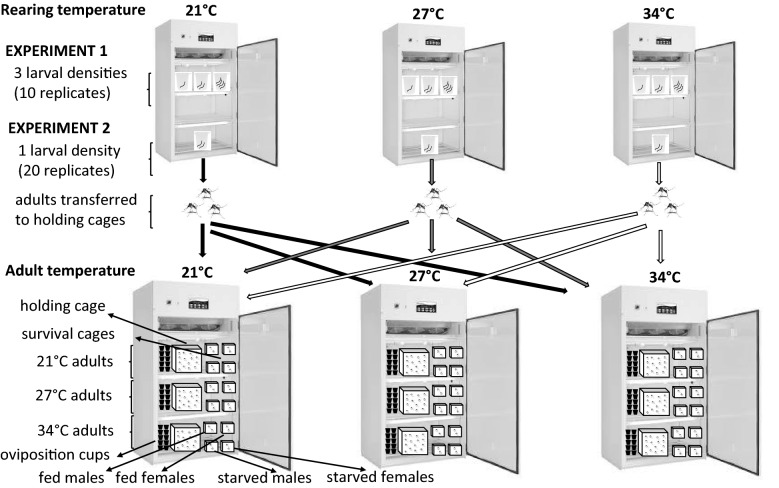
Schematic representation of the design for Experiments 1 and 2. Larvae allowed to develop at three temperatures (21 °C, 27 °C, and 34 °C) and emerging adults kept at the same temperature at which they were reared as juveniles, or placed at one of the other two temperatures. Densities for Experiment 1 varied (10, 20 and 40 individuals); however, only one density was used in Experiment 2 (20 individuals)

After emergence, adults from experimental units of each larval temperature (hereafter, source temperature) were pooled (yielding a possible 400 adults per temperature) and introduced into separate adult holding cages, placed in each of the three environmental chambers. The temperatures in these chambers (i.e. 20 °C, 27 °C and 34 °C) served as adult holding temperatures (hereafter, adult temperatures, see Fig. 1). This arrangement allowed the distinction between the effects of larval rearing and adult holding temperatures on female fecundity and adult survival. Each chamber had 3 cages, with each cage holding males and females from the same source temperature. The adults were provided with 10% sugar solution and held for 10 days to allow for enough interaction time for mating as well as minimize any age-dependent effects on mating.

For the fecundity assessment, female mosquitoes were blood-fed on an immobilized Japanese quail *Coturnix japonica* (IACUC 11092207) on day 11. Afterwards, females were allowed one week to complete egg maturation after which time 10 individuals were randomly selected from each cage and placed individually in oviposition sites made up of 600 ml black plastic cups containing 200 ml of gravid water (from tire inoculum), lined with paper towel to serve as an oviposition substrate and covered with no-see-um mesh. Each cup was provided with cotton pads soaked in 10% sugar solution for adult female sustenance. After 5 days females were removed from oviposition cups, freeze-killed and dried for 48 h at 50 °C, after which their dry weights were measured using the XP2U ultra-microbalance (Mettler Toledo Inc., Columbus, Ohio). Eggs laid per cup were removed, counted, and subsequently hatched simultaneously using the procedure previously described.

For adult survival assessment, 20 females from each of the 3 cages for each source temperature were randomly selected to be held under two conditions in the same environmental chamber. For each condition (starved, fed), 10 females were placed together in individual cages and provided with soaked cotton pads. In the starved-condition, adults received cotton pads soaked with RO water to avoid dehydration whereas for the fed-condition mosquitoes received pads soaked in 10% sugar solution. All cage positions were rotated in a clockwise fashion within the environmental chambers every 24 h to control for effects of location within the chamber. Cages were also inspected every two days for adult survival. Dead adults were removed when observed, identified to sex, and recorded. This experiment was allowed to run for 140 days to allow ample time for mosquito survival across the three adult temperatures.

### Statistical analyses

Before conducting statistical analyses for both experiments, we tested the entire dataset for statistical assumptions of normality and homogeneity of variances. Development time, adult mass, and adult survivorship data met assumptions. However, larval survival and female fecundity (number of eggs laid) were transformed using arcsine square root transformation and square root +1 transformation, respectively, to meet assumptions. We used multivariate analysis of variance (MANOVA) to test the effects of larval rearing temperature, density ratios, and their interaction on development time and emerging adult dry mass. Standardized canonical coefficients (SCC) were also used to indicate the important variables accounting for observed multivariate effects as well as their relationship to each other, e.g. positive or negative [31]. We also used analysis of variance (ANOVA) to test the effects of larval rearing temperature, density ratios, and their interaction on larval survival. Differences in survival among temperature and density interactions were identified using the Tukey-Kramer HSD *post-hoc* test for multiple comparisons [32]. For adult performance with larval and adult environmental temperature variations, we used a two-way analysis of covariance (ANCOVA) with female mass as the covariate to test the null hypothesis that female fecundity is highest when larval and adult temperatures are the same. Differences in survival among temperature interaction were identified using the Tukey-Kramer HSD *post-hoc* test for multiple comparisons [32]. We also used the Kaplan-Meier Survival Analysis and Mantel-Cox Log-Rank tests to test the null hypothesis of no change in survival across adult environmental temperatures for individuals from different larval environmental temperatures. Statistical analyzed were conducted using JMP® Version 10 [33].

## Results

### Experiment 1: Temperature and larval competition

**Fig. 2:**
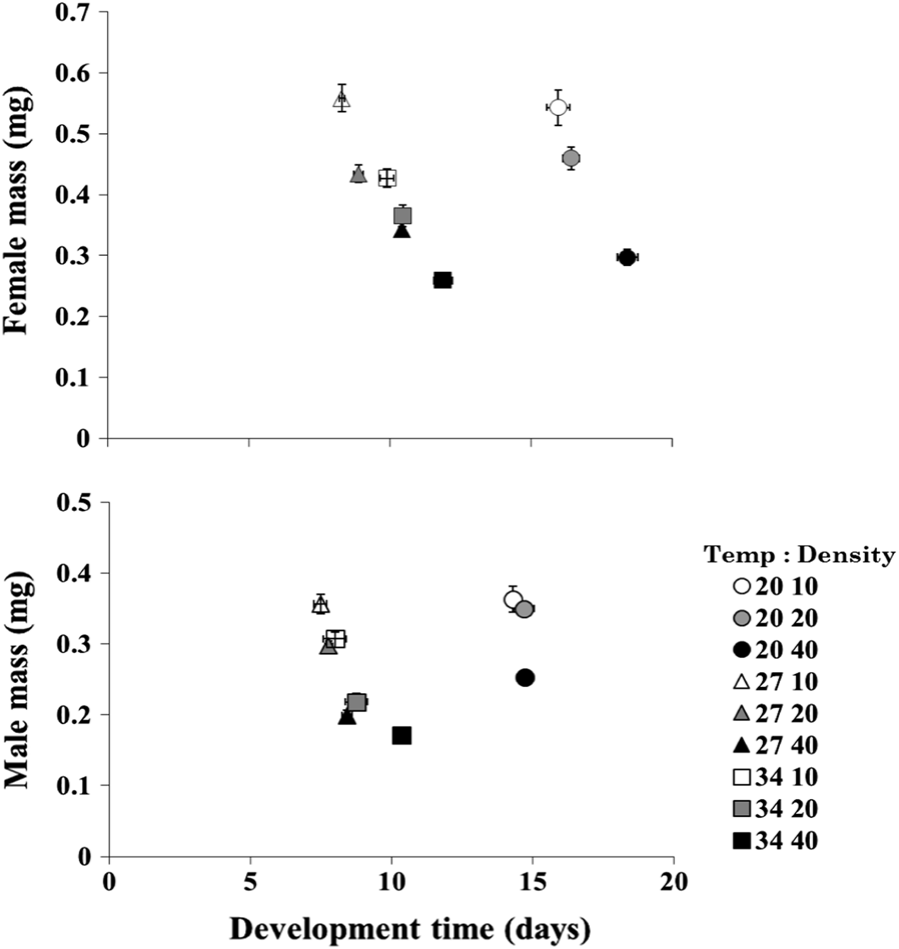
Bi-plots of means (±SE) for mass and development times for male and female *Ae. albopictus* reared across different environmental temperature and larval density combinations

There were significant effects of temperature, larval density, and their interaction on competitive outcomes (development time and adult mass) for male and female *Ae. albopictus.* Standardized canonical coefficients (SSC) indicated that adult mass and development time for males contributed more to the significant MANOVA effect compared to mass and development time for females (Table 1). Mass for both males and females decreased with increasing density, and were lowest at all temperatures under high density combinations (temperature:density, 20:40, 27:40 and 34:40) and highest at all temperatures under low density combinations (20:10, 27:10 and 34:10). Increasing the larval rearing temperature from 20 °C to 27 °C resulted in no difference in male and female mass. However, the increase from 27 °C to 34 °C produced a significant reduction in adult mass for males and females (Fig. 2).

**Table 1:** Multivariate ANOVA for main effects and multivariate pairwise contrasts of temperature and larval density effects on male and female *Aedes albopictus* development time and adult mass (shown in bold). Significant contributors to multivariate effects are shown in bold type

Factor	*df*	Pillai’s trace	*F*-value	*P*-value	Standardized canonical coefficients			
					Female		Male	
					Development time	Mass	Development time	Mass
Density	8, 148	0.86	14.06	**<0.001**	1.30	-1.16	0.37	-1.12
Temperature	8, 148	1.48	23.30	**<0.001**	3.06	0.02	0.02	1.03
Density × temperature	16, 304	0.57	3.13	**<0.001**	1.28	1.60	**1.94**	**2.48**

Development time for males and females increased with decreasing temperatures, and was significantly longer at 20 °C compared to 27 °C and 34 °C. There were no significant differences in male development time with larval density at 20 °C and 27 °C. However, at 34 °C, an increase in larval density from 20 to 40 individuals resulted in a significant increase in male development time. Similarly, an increase in larval density from 20 to 40 individuals resulted in a significant increase in female development time across all larval temperatures (Fig. 2).

For larval survival, there were no effects of temperature (*F*_(2,86)_* = *1.444, *P = *0.2421), larval density (*F*_(2,86)_* = *0.584, *P = *0.5601) or their interaction (*F*_(4,86)_* = *1.532, *P = *0.2006). No pupae were produced after day 29 and no new adults emerged, and all remaining larvae died before day 60, and thus the run time appeared sufficient.

### Experiment 2: Adult performance with larval and adult environmental temperatures

**Fig. 3:**
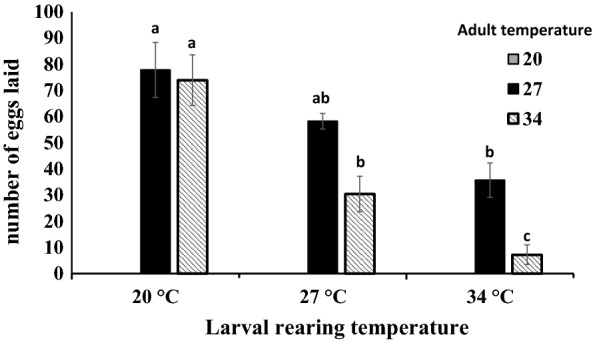
Mean (± SE) fecundity of female *Ae. albopictus* across three adult temperatures reared from the same temperatures at their larval stage. Different lowercase letters indicate significant pairwise differences between fecundity within adult environmental temperature

The number of eggs laid differed significantly with adult temperature (*F*_(2,36)_* = *13.6113, *P < *0.001), among larval rearing temperature (*F*_(2,36)_* = *22.8228, *P < *0.001), and their interaction (*F*_(4,36)_* = *3.6110, *P = *0.0337) (Fig. 3); we found no significant effects of the covariate (female mass) by itself or in interactions with adult or larval rearing temperature (*P* > 0.05). Under the adult temperature of 20 °C, no eggs were oviposited by females from any larval temperature. At the adult temperature of 27 °C, fewer eggs were produced in the 34 °C compared with the 20 °C larval rearing temperature. However, at the adult temperature of 34 °C, female fecundity significantly decreased across all larval temperatures; adult females from low larval rearing temperatures laid the highest number of eggs whereas those from high larval rearing temperatures laid the fewest (Fig. 3).

**Fig. 4:**
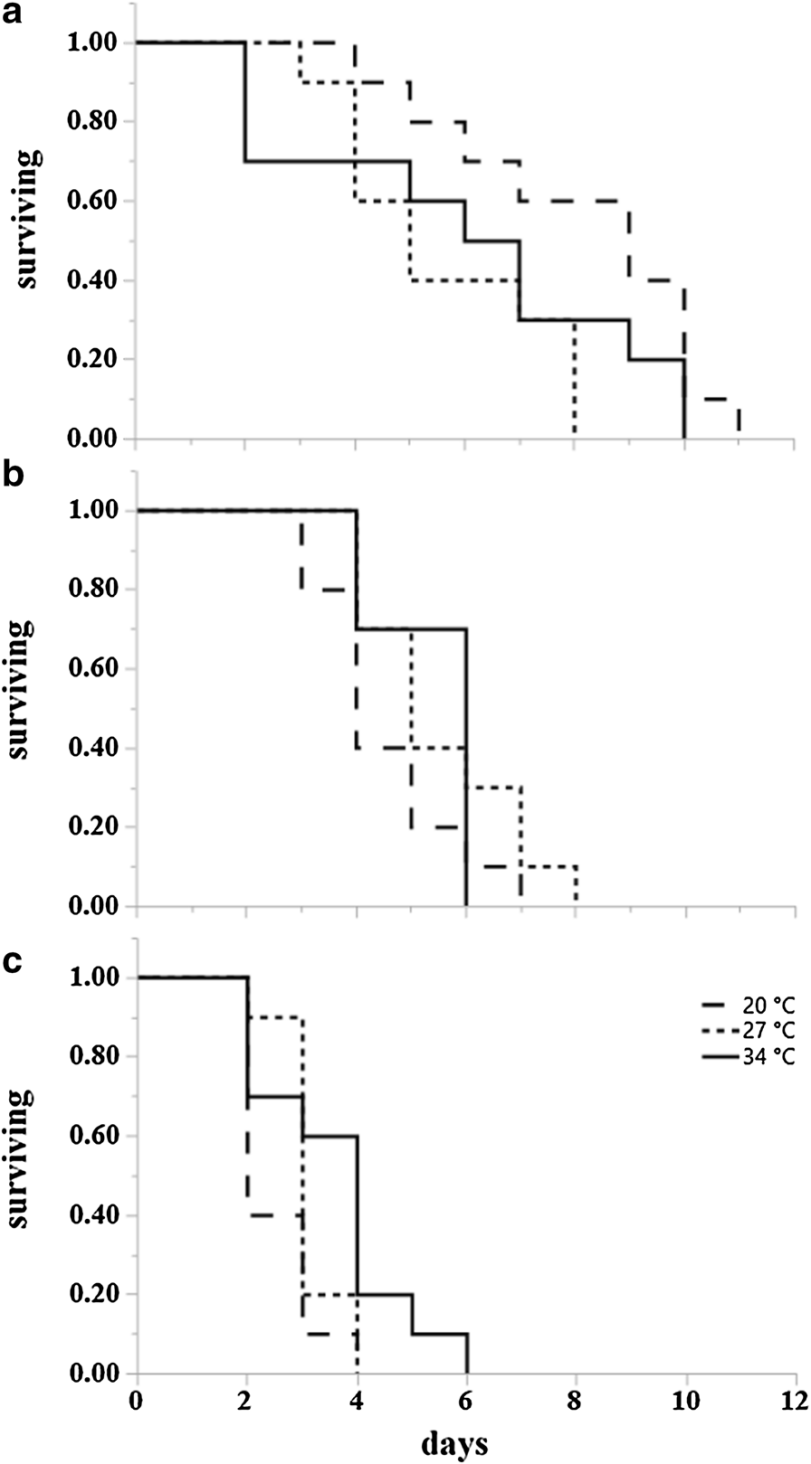
Kaplan-Meier survival plots of starved *Ae. albopictus* males at three temperatures (21 °C, 27 °C and 34 °C) after rearing as larvae at the same three temperatures. **a** Survival curves at 20 °C for individuals from larval rearing temperature 20 °C (dashed), 27 °C (dotted) and 34 °C (solid). **b** Survival curves at 27 °C. **c** Survival curves at 34 °C

**Fig. 5:**
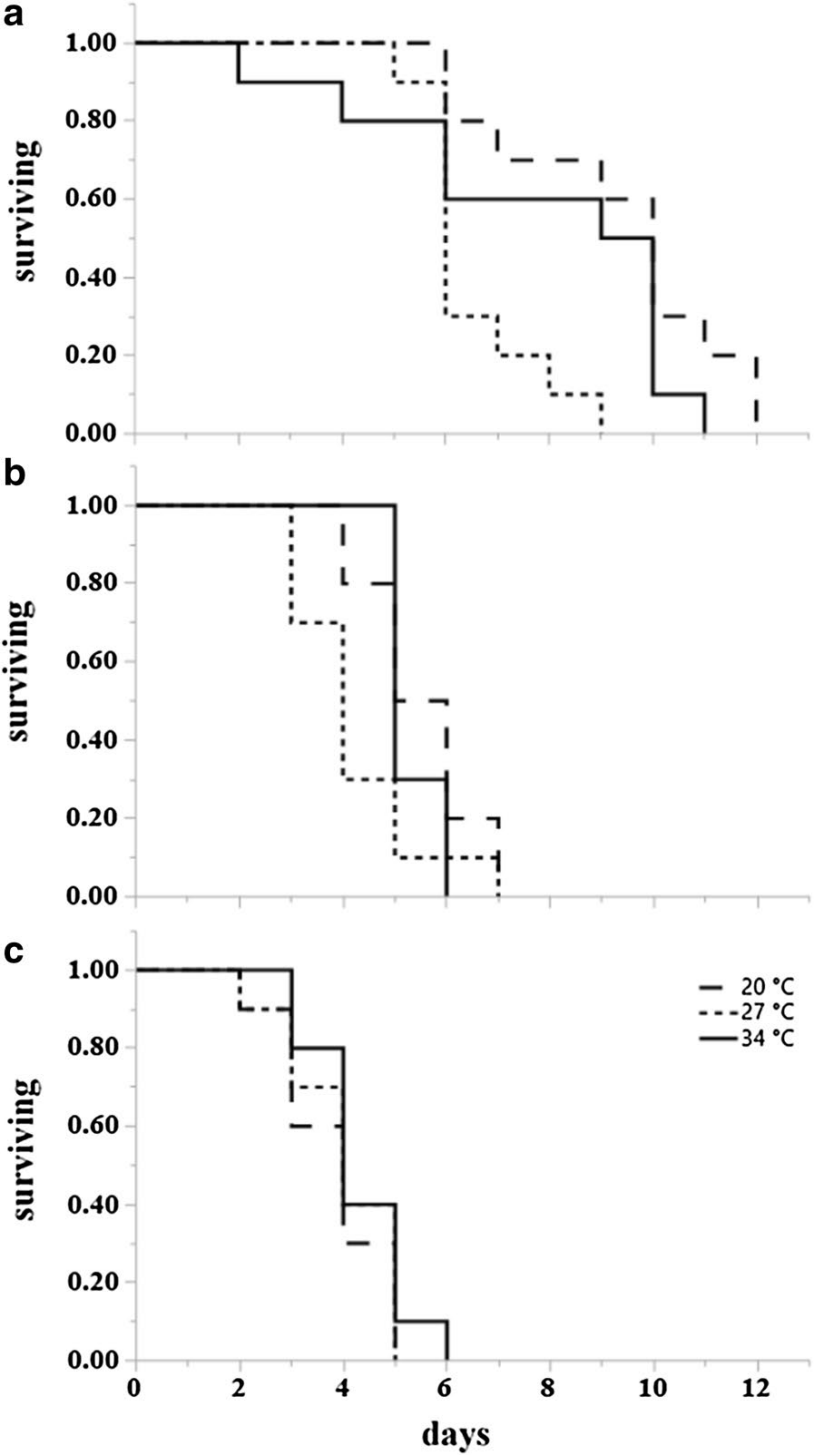
Kaplan-Meier survival plots of starved *Ae. albopictus* females at three temperatures (21 °C, 27 °C and 34 °C) after rearing as larvae at the same three temperatures. **a** Survival curves at 20 °C for individuals from larval rearing temperature 20 °C (dashed), 27 °C (dotted) and 34 °C (solid). **b** Survival curves at 27 °C. **c** Survival curves at 34 °C

**Fig. 6:**
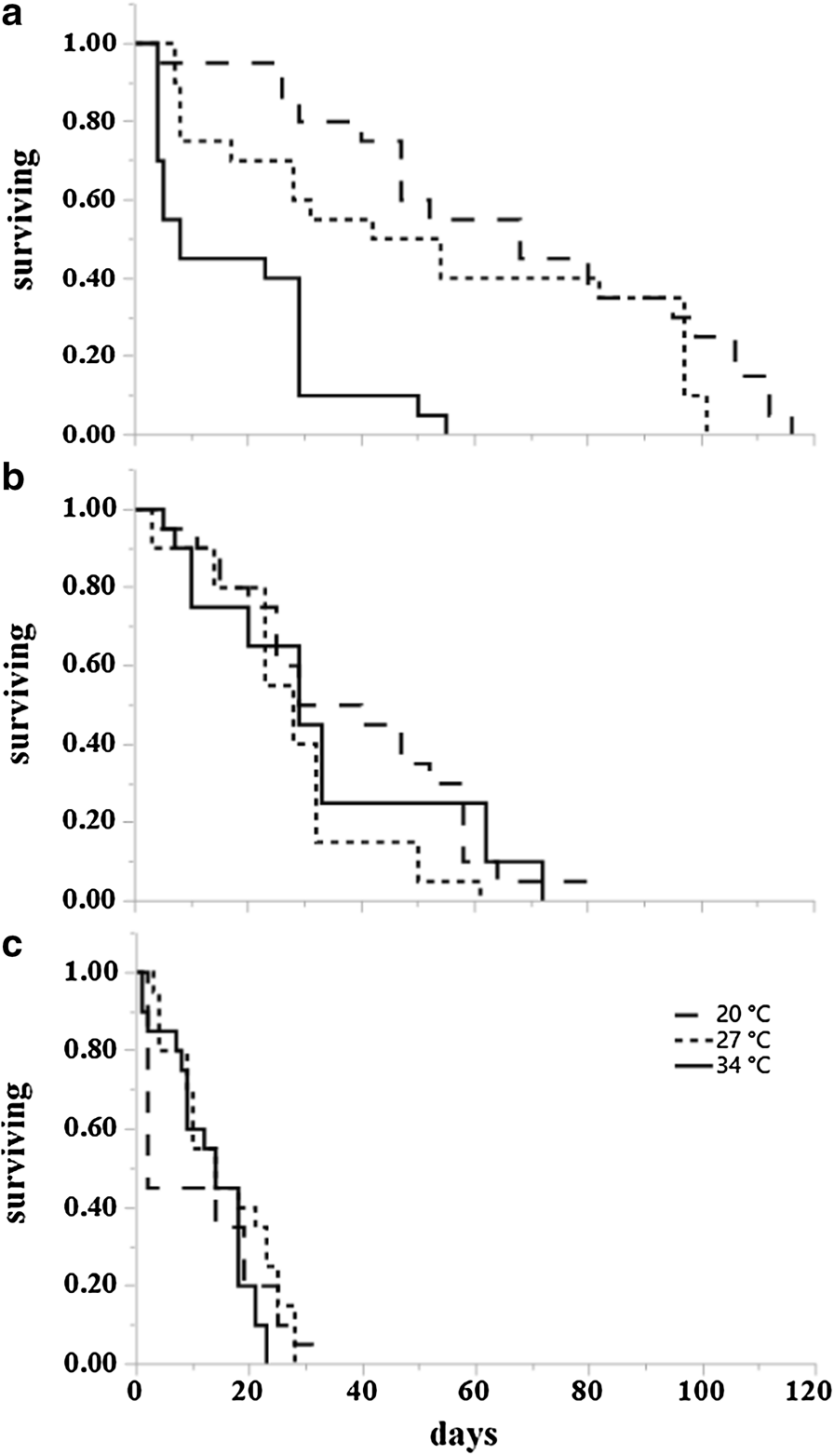
Kaplan-Meier survival plots of fed *Ae. albopictus* males at three temperatures (21 °C, 27 °C and 34 °C) after rearing as larvae at the same three temperatures. **a** Survival curves at 20 °C for individuals from larval rearing temperature 20 °C (dashed), 27 °C (dotted) and 34 °C (solid). **b** Survival curves at 27 °C. **c** Survival curves at 34 °C

**Fig. 7:**
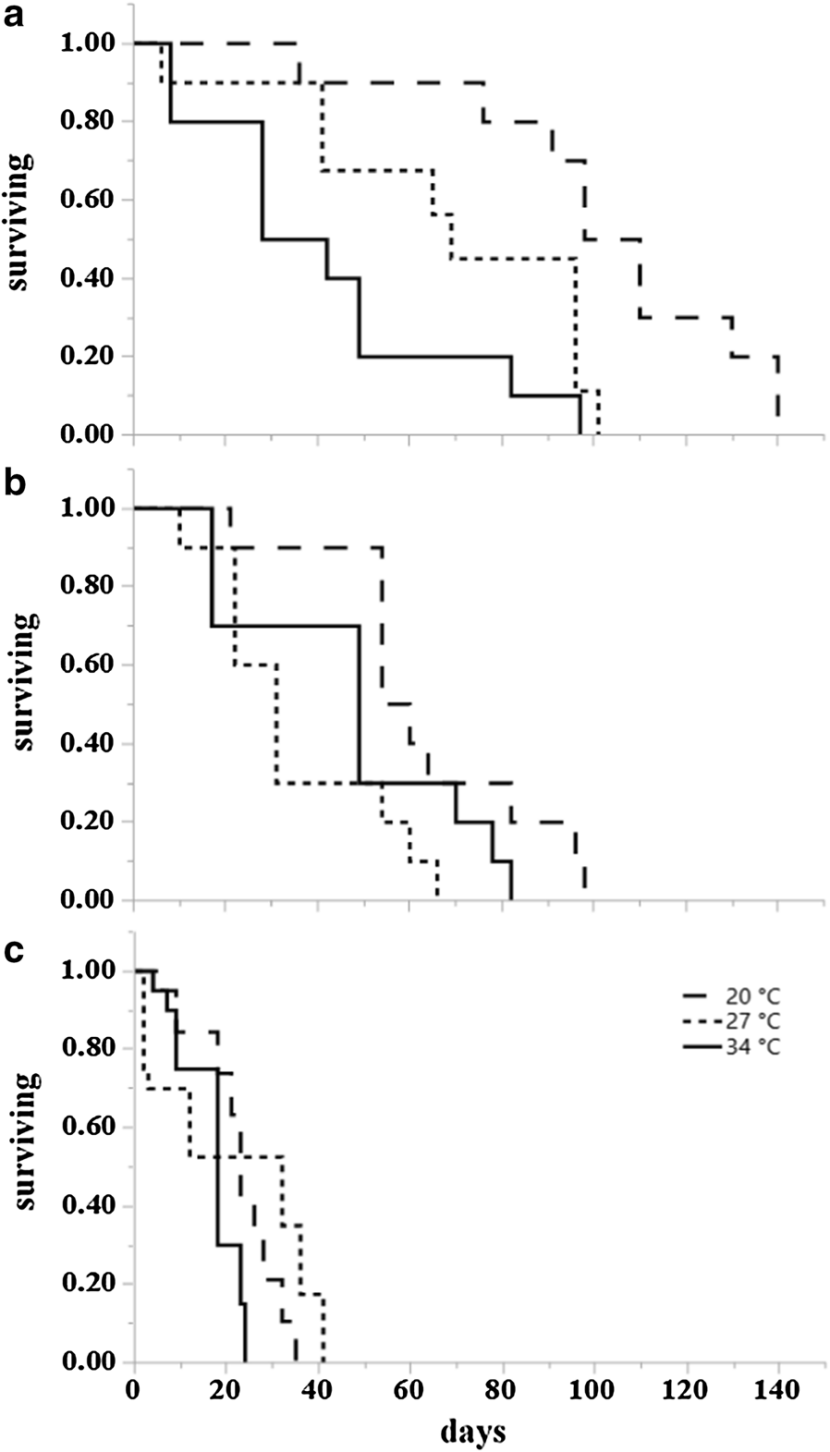
Kaplan-Meier survival plots of fed *Ae. albopictus* females at three temperatures (21 °C, 27 °C and 34 °C) after rearing as larvae at the same three temperatures. **a** Survival curves at 20 °C for individuals from larval rearing temperature 20 °C (dashed), 27 °C (dotted), 34 °C (solid). **b** Survival curves at 27 °C. **c** Survival curves at 34 °C

Results from survival analysis (Table 2) showed that at 20 °C adult temperature, the overall influence of larval rearing temperatures was significant for starved males (*P = *0.0461) (Fig. 4a), starved females (*P = *0.0054) (Fig. 5a), fed males (*P < *0.0001) (Figs. 3, 4a, 5a) and fed females (*P = *0.0008) (Fig. 6a). Specifically, at 20 °C adults from the 20 °C larval rearing temperature survived longer than those from 27 °C and 34 °C, irrespective of their starved or fed condition. The overall effect of larval rearing temperature on adult survival at 27 °C was significant only for starved females (*P = *0.0274) (Fig. 5b) and fed females (*P = *0.0341) (Fig. 7a). Just like in the adult rearing temperature of 20 °C, adults from the 20 °C larval rearing temperature survived longer than those from either 27 °C or 34 °C. At 34 °C the overall effect of larval rearing temperature on adult survival was only significant in starved males (*P = *0.0302). In this case however, adults from the 34 °C larval rearing temperature survived longer than those from 20 °C and 27 °C (Fig. 4c).

**Table 2:** Log rank test results on the effect of larval environmental temperatures on the survival of starved and fed adult *Aedes albopictus*, at three adult environmental temperatures. Significant effects for survival curves are shown in bold type

Condition	Adult temperature (°C)	Males			Females		
		*χ* ^2^	*df*	*P*-value	Log rank	*df*	*P*-value
Starved	20	6.1530	2	**0.0461**	10.4340	2	**0.0054**
Starved	27	3.5478	2	0.1697	7.1952	2	**0.0274**
Starved	34	7.0016	2	**0.0302**	1.0502	2	0.5915
Fed	20	26.4959	2	<**0.0001**	14.3655	2	**0.0008**
Fed	27	4.2224	2	0.1211	6.7581	2	**0.0341**
Fed	34	2.2646	2	0.3223	1.0487	2	0.5920

## Discussion

We found support for our hypothesis that larval environmental temperature alters the intensity of density-dependent intraspecific larval competition in *Ae. albopictus*. As predicted, there was an interaction between density and temperature such that development time and emerging adult body mass decreased with increasing temperature and density. Mosquitoes had their lowest body mass at 34 °C with 40 individuals and the highest mass at 20 °C with 10 individuals. Density alone also affected body mass, with higher densities producing smaller males and females. These findings are in line with results from other studies that consider density-dependent competition for food during larval stages as one of the most important factors affecting mosquito population dynamics [34, 35]. Increasing temperature from 20 °C to 27 °C resulted in no significant change in body mass, although body mass decreased with increase in temperature from 27 °C to 34 °C. Thus, density-dependent competition and larval temperature could play an important role in shaping the overall size of adult populations of mosquitoes and other invertebrates with complex life histories.

Development time to adulthood was longer with decreasing temperatures for both males and females. Development time was generally shorter for males than females, most likely due to trade-off associated lower nutritional thresholds for males of most mosquito species. Shorter development time in males may also result from protandry, a form of sexual selection whereby males sacrifice mass to develop faster for access to virgin females, who take longer to develop to increase their mass and life-time fecundity [36]. Protandry has been reported in *Ae. albopictus* [37]. Because larvae took longer to develop at cooler temperatures, larger adults were produced and there were wider differences in development time at the lowest temperature, than at intermediate and higher temperatures. These results lend support to findings that suggest that increased temperature is generally associated with shorter development time and smaller adults in numerous aquatic taxa (reviewed in [7, 30]) and for many mosquito species [9, 38–43]. Taken together, these results point to the conclusion that in addition to other measures of larval performance in mosquito life history, the size of emerging adults and the larval development time can be influenced by density-dependent competition and temperature experienced during larval development.

Because there was some evidence that temperature and density may interact to influence larval survival in mosquitoes [40, 44], we expected decreased survivorship under hotter, denser situations. However, unlike their effects on development time and mass, temperature and density had no effect on larval survival in *Ae. albopictus*. This lack of effect was not altogether surprising given that other studies have been unable to identify differences in survivorship across similar larval temperature ranges [43, 45] and densities [46]. One possible reason for this is that the temperature and density combinations considered in this study as well as in others did not span a wide enough gradient to impose substantial stress that induced higher mortality in *Ae. albopictus*, even though they may have been significant enough to alter *Ae. albopictus* metabolic rates and hence development time and adult mass.

Based on the premise of acclimation and carry-over effects of fitness advantage across life stages, we expected fecundity to be greatest when larval and adult temperatures were similar. To the contrary, we found this to be true only at temperature of 27 °C. At the adult temperature of 20 °C, no eggs were laid by females from all larval rearing temperatures. One possible explanation for this is the well supported diapause mechanism in adults may have prevented or delayed egg deposition; however, egg diapause often manifests at temperatures lower than 20 °C [47]. It is also possible that the females exposed to this temperature were unmated and so could not produce mature eggs; we did not physically observe all females copulate nor did we dissect spermathecae or ovaries to confirm insemination and egg development in the blood-fed females. Moreover, because all females were only allowed five days to oviposit following a one-week egg maturation period, those at 20 °C may have been less active and delayed oviposition such that there was not enough time elapsed for females to oviposit if they were mated and developed eggs. Future experiments could look into extending the period of exposure thereby allowing individual females ample time to lay eggs, and subsequently dissecting them to determine stages of egg development. Another possibility that may require future examination is the extent to which male fertility may have been negatively impacted by low temperatures [48].

At 27 °C, there was no significant difference in the number of eggs oviposited by females based on any of the three larval rearing temperatures. It is specifically at this temperature that females from each larval environmental temperature oviposited the highest number of eggs. Based on this, we can conclude that 27 °C is close to the optimum temperature required for *Ae. albopictus* intrinsic rate of growth (*r*) which lies between 25–30 °C [44]. However, at 34 °C, mosquitoes laid significantly fewer eggs than those at 27 °C. Females subjected to 20 °C laid more eggs but this was not significantly different from those laid by females in 27 °C. The disparity in fecundity at higher temperatures could be as a result of a fitness advantage or trade-off associated with development at the larval temperatures. Fecundity-size relationships suggest that females from lower larval rearing temperatures would be larger and more fecund, whereas those from higher larval rearing temperatures would be smaller and less fecund [18]. It appears likely that the effect of larval rearing temperature could be carried over through female size to affect fecundity. In this study however, we controlled for female body size on the number of eggs to test for carry-over effects of larval rearing temperature alone on female fecundity at adult temperatures. Based on these results, we can conclude that higher larval-adult temperatures lead to greater differences in female fecundity. The implication is that in the absence of differences in body size, other factors such as development time, rate of metabolic and foraging activity, and resource allocation could be contributing factors to the observed trend under the high adult temperature. Moreover, higher temperatures could be detrimental for mosquito reproduction and may result in activation of heat-shock proteins [49, 50] that may enhance thermo-tolerance and reduce protein denaturation.

In our experiment on adult performance with larval and adult temperatures, we hypothesized that adult performance would vary with interactions between larval habitat temperature and adult habitat temperature, and we predicted that survival would be highest when larval and adult habitat temperatures were similar. Results from this experiment showed that adults of both sexes subjected to cooler temperatures had higher survival relative to those under warmer temperatures regardless of the rearing temperature of the immature stages (20 °C > 27 °C > 34 °C) and adult condition (starved or fed). This was the same outcome as in a similar study by Alto & Betinardi [22]; these authors suggested the possibility of no buffering effect of cool larval habitat temperature against the deleterious effects of warm conditions of the adults, and that adult survival may not be influenced by temperature experienced during the larval stage. Because larval rearing temperature has been shown to influence the phenotypic traits of adults, such as reproduction [41] and susceptibility to infection [43], it is possible that longer development times associated with low larval rearing temperatures facilitate the production of large-sized adults because of greater nutrient uptake and energy reserves at adult emergence [41]. Higher fecundity at lower temperatures appears to bolster the notion that slower developing females are larger, and similar effects of lower larval temperatures perhaps can be extended to adult survival patterns. Unlike Alto & Betinardi [22], our earlier experiment on temperature and larval competition produced larger adults from lower temperatures. This is evidence of larval temperature effects on adult mass and with it, we can conclude that low temperatures during the immature stages affects adult survival by producing adults with increased size. These adults could potentially have acquired high teneral reserves, and a higher probability of adult survival [51, 52].

Mosquitoes, being poikilothermic organisms, are susceptible to external temperature variations that directly influences their body temperature [53]. This study was designed as a general test of the effects of drastic temperature shifts acting on the immature and adult stages and consequences on life-history performance. Our results clearly show that larval and adult temperatures interact to affect female fecundity in *Ae. albopictus*, and represents the first study of its kind to our knowledge. Our experimental design did not take into account temperature fluctuations and daily temperature ranges, factors that are known to influence disease transmission and are important in predictive models and control efforts [54, 55]. In nature, immature mosquitoes are subject to daily fluctuations in water temperature during development and adults experience similar changes in air temperature. Previous studies have suggested that fluctuating diel temperatures, rather than constant temperatures, are a better measure of performance [56–59]. Murdock et al. [58] found that expression of genes in the mosquito *Anopheles stephensi* involved in immunity were different under constant *versus* diurnal temperature fluctuations, and that time of day of infection interacted with fluctuating temperatures to produce complex outcomes in immunity. More work is clearly needed to understand how variation in temperature affects patterns in life history and immunity in mosquitoes [57]; however, our study suggests that carry-over effects occur even under constant temperatures, and have important implications for populations and life history of *Ae. albopictus*.

Our work also serves to further add to the growing body of knowledge about the role of changing climates on ectotherms, especially insects [60]. Yee et al. [16] have shown that populations of *Ae. albopictus* are negatively affected by predicted future warming; however, that study did not consider carry-over effects. More broadly, increasing temperatures due to climate change would likely have deleterious consequences for tropical insects, who are relatively sensitive to temperature change and currently live very close to their optimal temperature [60]. Conversely, climate change may enhance the fitness of temperate species living in climates below their physiological optima and broader thermal tolerances [60]. Our results suggest that temperature can have unpredictable effects on adults when other life-history stages have a different thermal experience. Such experiences can indeed alter the way in which climate change may affect animals during growth which can result in carry-over effects on morphology that affect adult performance [61]. Going forward, it is hard to predict if other species of ectotherms generally or mosquitoes specifically will respond in similar ways as *Ae. albopictus*. However, our study has further highlighted the importance of temperature as an influence on how adult phenotypes are the product of shared larval and adult experiences.

## Conclusions

We show that temperature affects life history of developing larvae under competitive interactions and can also alter adult fitness traits such as fecundity as the disparity between larval and adult temperatures increases. These carry-over effects, from larvae to adult, can have importance for our understanding of mosquito immunity and pathogen transmission, and for how different life-history stages of *Ae. albopictus* and other vectors of disease may respond to changing climates.

## Abbreviations

RO: reverse osmosis
MANOVA: Multivariate Analysis of Variance
ANOVA: Analysis of Variance

## References

[1] Kingsolver JG, Woods HA, Buckley LB, Potter KA, MacLean HJ, Higgins JK (2011). Complex life cycles and the responses of insects to climate change. Int Comp Biol..

[2] Green BS, McCormick MI (2005). Maternal and paternal influences determine size, growth and performance in tropical reef fish larvae. Mar Ecol Prog Ser..

[3] McCormick MI, Gagliano M. Carry-over affects-the importance of a good start. In: Proc 11th Int Coral Reef Sym Ses. 2008; vol 10, p. 305–10.

[4] Kingsolver JG, Huey RB (2008). Size, temperature, and fitness: three rules. Evol Ecol Res..

[5] Steinwascher K (1982). Relationship between pupal mass and adult survivorship and fecundity for *Aedes aegypti*. Environ Entomol..

[6] Day JF, Ramsey AM, Zhang J-T (1990). Environmentally mediated seasonal variation in mosquito body size. Environ Entomol..

[7] Harrison Blount JD, Inger R, Norris DR, Bearhop S (2011). Carry-over effects as drivers of fitness differences in animals. J Anim Ecol..

[8] Parker BM (1986). Hatchability of eggs of *Aedes taeniorhynchus* (Diptera: Culicidae): effects of different temperatures and photoperiods during embryogenesis. Ann Entomol Soc Am..

[9] Rueda LM, Patel KJ, Axtell RC, Stinner RE (1990). Temperature-dependent development and survival rates of *Culex quinquefasciatus* and *Aedes aegypti* (Diptera: Culicidae). J Med Entomol..

[10] Crans WJ, Sprenger DA, Mahmod F (1996). The blood-feeding habits of *Aedes sollicitans* (Walker) in relation to Eastern Equine Encephalitis virus in coastal areas of New Jersey, II. Results of experiments with caged mosquitoes and the effects of temperature and physiological age on host selection. J Vect Ecol..

[11] Hurlbut HS (1973). The effect of environmental temperature upon the transmission of St. Louis encephalitis virus by *Culex pipiens quinquefasciatus*. J Med Entomol..

[12] Barreaux Stone CM, Barreaux P, Koella JC (2018). The relationship between size and longevity of the malaria vector *Anopheles gambiae* (*s.s.*) depends on the larval environment. Parasit Vectors.

[13] Adelman ZN, Anderson MA, Wiley MR, Murreddu MG, Samuel GH, Morazzani EM, Myles KM (2013). Cooler temperatures destabilize RNA interference and increase susceptibility of disease vector mosquitoes to viral infection. PLoS Negl Trop Dis..

[14] Mordecai EA, Paaijmans KP, Johnson LR, Bazler C, Ben-Horin T, de Moor E (2013). Optimal temperature for malaria transmission is dramatically lower than previously predicted. Ecol Lett..

[15] Padmanabha H, Bolker B, Lord CC, Rubio C, Lounibos LP (2011). Food availability alters the effects of larval temperature on *Aedes aegypti* growth. J Med Entomol..

[16] Yee DA, Ezeakacha NF, Abbott K (2016). The interactive effects of photoperiod and future climate change may have negative consequences for a wide-spread invasive insect. Oikos..

[17] Moller-Jacobs LL, Murdock CC, Thomas MB (2014). Capacity of mosquitoes to transmit malaria depends on larval environment. Parasit Vectors..

[18] Sibly RM, Atkinson D (1994). How rearing temperature affects optimal adult size in ectotherms. Funct Ecol..

[19] Telang A, Qayum AA, Parker A, Sacchetta BR, Byrnes GR (2012). Larval nutritional stress affects vector immune traits in adult yellow fever mosquito *Aedes aegypti* (*Stegomyia aegypti*). Med Vet Entomol..

[20] Reiskind MH, Lounibos LP (2009). Effects of intraspecific larval competition on adult longevity in the mosquitoes *Aedes aegypti* and *Aedes albopictus*. Med Vet Entomol..

[21] Muturi EJ, Allan BF, Ricci J (2012). Influence of leaf detritus type on production and longevity of container-breeding mosquitoes. Environ Entomol..

[22] Alto BW, Bettinardi D (2013). Temperature and dengue virus infection in mosquitoes: independent effects on the immature and adult stages. Am J Trop Med Hyg..

[23] Kay BH, Fanning ID, Mottram P (1989). Rearing temperature influences flavivirus vector competence of mosquitoes. Med Vet Entomol..

[24] Brubaker JF, Turell MJ (1998). Effect of environmental temperature on the susceptibility of *Culex pipiens* (Diptera: Culicidae) to Rift Valley Fever virus. J Med Entomol..

[25] Christiansen-Jucht C, Parham PE, Saddler A, Koella JC, Basáñez MG (2014). Temperature during larval development and adult maintenance influences the survival of *Anopheles gambiae s.s*. Parasit Vectors..

[26] Westby KM, Juliano SA (2015). Simulated seasonal photoperiods and fluctuating temperatures have limited effects on blood feeding and life history in *Aedes triseriatus* (Diptera: Culicidae). J Med Entomol..

[27] Lounibos LP (2002). Invasions by insect vectors of human disease. Annu Rev Entomol..

[28] Nawrocki SJ, Hawley WA (1987). Estimation of the northern limits of distribution of *Aedes albopictus* in North America. J Am Mosq Cont Assoc..

[29] Alto BW, Juliano SA (2001). Temperature effects on the dynamics of *Aedes albopictus* (Diptera: Culicidae) populations in the laboratory. J Med Entomol..

[30] Atkinson D (1995). Effects of temperature on the size of aquatic ectotherms: exceptions to the general rule. J Ther Biol..

[31] Scheiner SM, Scheiner SM, Gurevitch J (2001). MANOVA. Multiple response variables and multi species interactions. Design and analysis of ecological experiments.

[32] Tukey JW (1991). The philosophy of multiple comparisons. Stat Sci..

[33] SAS Institute Inc. (2012). Using JMP 10.

[34] Moore CG, Fisher BR (1969). Competition in mosquitoes. Density and species ratio effects on growth, mortality, fecundity, and production of growth retardant. Ann Entomol Soc Am..

[35] Gilles JR, Lees RS, Soliban SM, Benedict MQ (2001). Density-dependent effects in experimental larval populations of *Anopheles arabiensis* (Diptera: Culicidae) can be negative, neutral, or overcompensatory depending on density and diet levels. J Med Entomol..

[36] Kleckner CA, Hawley WA, Bradshaw WE, Holzapfel CM, Fisher IJ (1995). Protandry in *Aedes sierrensis*: the significance of temporal variation in female fecundity. Ecology..

[37] Yee DA, Juliano SA, Vamosi SM (2012). Seasonal photoperiods alter developmental time and mass of an invasive mosquito, *Aedes albopictus* (Diptera: Culicidae), across its north-south range in the United States. J Med Entomol..

[38] Heuvel MJ (1963). The effect of rearing temperature on the wing length, thorax length, leg length and ovariole number of the adult mosquito, *Aedes aegypti* (L.). Trans R Entomol Soc Lon..

[39] Brust RA (1967). Weight and development time of different stadia of mosquitoes reared at various constant temperatures. Can Entomol..

[40] Lyimo EO, Takken W, Koella JC (1992). Effect of rearing temperature and larval density on larval survival, age at pupation and adult size of *Anopheles gambiae*. Entomol Exp Appl..

[41] Briegel H, Timmermann SE (2001). *Aedes albopictus* (Diptera: Culicidae): physiological aspects of development and reproduction. J Med Entomol..

[42] Ragland GJ, Kingsolver JG (2008). The effect of fluctuating temperatures on ectotherm life-history traits: comparisons among geographic populations of *Wyeomyia smithii*. Evol Ecol Res..

[43] Westbrook CJ, Resikind MH, Rsko KN, Greene KE, Lounibos LP (2010). Larval environmental temperature and the susceptibility of *Aedes albopictus* Skuse (Diptera: Culicidae) to chikungunya virus. Vector Borne Zoonotic Dis..

[44] Delatte H, Gimonneau G, Triboire A, Fontenille D (2009). Influence of temperature on immature development, survival, longevity, fecundity, and gonotrophic cycles of *Aedes albopictus*, vector of chikungunya and dengue in the Indian Ocean. J Med Entomol..

[45] Muturi EJ, Lampman L, Costanzo C, Alto BW (2011). Effect of temperature and insecticide stress on life-history traits of *Culex restuans* and *Aedes albopictus* (Diptera: Culicidae). J Med Entomol..

[46] Yoshioka MJ, Couret J, Kim F, McMillan J, Burkot TR, Dotson EM, Kitron U (2012). Diet and density dependent competition affect larval performance and oviposition site selection in the mosquito species *Aedes albopictus* (Diptera: Culicidae). Parasit Vectors..

[47] Armbruster P (2016). Photoperiodic diapause and the establishment of *Aedes albopictus* (Diptera: Culicidae) in North America. J Med Entomol..

[48] Carrington LB, Armijos MV, Lambrechts L, Barker CM, Scott TW (2013). Effects of fluctuating daily temperatures at critical thermal extremes on *Aedes aegypti* life-history traits. PLoS One.

[49] Feder ME (1999). Organismal, ecological, and evolutionary aspects of heat-shock proteins and the stress response: established conclusions and unresolved issues. Am Zool..

[50] Rinehart JP, Hayward SAL, Elnitsky MA, Sandro LH, Lee RE, Denlinger DL (2006). Continuous up-regulation of heat shock proteins in larvae, but not adults, of a polar insect. Proc Natl Acad Sci USA.

[51] Hawley WA (1985). The effect of larval density on longevity of a mosquito, *Aedes sierrensis*: epidemiological consequences. J Anim Ecol..

[52] Nasci RS (1986). Relationship between adult mosquito (Diptera: Culicidae) body size and parity in field populations. Environ Entomol..

[53] Hawley WA (1988). The biology of *Aedes albopictus*. J Am Mosq Control Assoc Suppl..

[54] Paaijmans KP, Blanford S, Bell AS, Blanford JI, Read AF, Thomas MB (2010). Influence of climate on malaria transmission depends on daily temperature variation. Proc Natl Acad Sci USA.

[55] Lambrechts L, Paaijmans KP, Ransiri T, Carrington LB, Kramer LD, Thomas MB, Scott TW (2011). Impact of daily temperature fluctuations on dengue virus transmission by *Aedes aegypti*. Proc Natl Acad Sci USA.

[56] Bradshaw WE (1980). Thermoperiodism and the thermal environment of the pitcher-plant mosquito, *Wyeomyia smithii*. Oecologia..

[57] Murdock CC, Paaijmans KP, Cox-Foster D, Read AF, Thomas MB (2012). Rethinking vector immunology: the role of environmental temperature in shaping resistance. Nat Rev Microbiol..

[58] Murdock CC, Paaijmans KP, Bell AS, King JG, Hillyer JF, Read AF, Thomas MB (2012). Complex effects of temperature on mosquito immune function. Proc Biol Sci.

[59] Murdock CC, Moller-Jacobs LL, Thomas MB (2013). Complex environmental drivers of immunity and resistance in malaria mosquitoes. Proc Biol Sci.

[60] Deutsch CA, Tewksbury JJ, Huey RB, Sheldon KS, Ghalambor CK, Haak DC, Martin PR (2008). Impacts of climate warming on terrestrial ecotherms across latitude. Proc Natl Acad Sci USA.

[61] McCauley SJ, Hammond JI, Mabry KE (2018). Simulated climate change increases larval mortality, alters phenology, and affects flight morphology of a dragonfly. Ecosphere..

